# Serum metabolomic signatures predict clinical outcomes in advanced non-small cell lung cancer treated with pembrolizumab plus platinum-based chemotherapy

**DOI:** 10.3389/fimmu.2026.1770764

**Published:** 2026-05-18

**Authors:** Peter May, Christof Winter, Inga Hubrecht, Adrian Patenge, Selina Strathmeyer, Roland Geyer, Steffen Heelemann, Jan Stratmann, Seyer Safi, Henriette Klein, Folker Schneller, Florian Bassermann, Aaron Becker von Rose

**Affiliations:** 1 Department of Medicine III, TUM School of Medicine and Health, Technical University of Munich, Munich, Germany; 2 Department of Clinical Chemistry, TUM School of Medicine and Health, Technical University of Munich, Munich, Germany; 3 lifespin GmbH, Regensburg, Germany; 4 Department of Internal Medicine V, Pulmonology, Allergology, Intensive Care Medicine, Saarland University Hospital, Homburg, Germany; 5 Division of Thoracic Surgery, TUM School of Medicine and Health, Technical University of Munich, Munich, Germany; 6 TranslaTUM, Center for Translational Cancer Research, Technical University of Munich, Munich, Germany; 7 Deutsches Konsortium für Translationale Krebsforschung (DKTK), Heidelberg, Germany; 8 Bavarian Cancer Research Center (BZKF), Munich, Germany; 9 Department of Emergency Medicine, TUM School of Medicine and Health, Technical University of Munich, Munich, Germany

**Keywords:** biomarker, branched-chain amino acids, immunotherapy, metabolomics, NSCLC, phospholipids

## Abstract

**Background:**

Predicting response to pembrolizumab plus chemotherapy in advanced non-small cell lung cancer (NSCLC) remains challenging. Serum metabolomics offers a promising approach to identify biomarkers capturing host–tumor metabolic interactions.

**Methods:**

We conducted nuclear magnetic resonance (NMR) spectroscopy-based metabolomic analysis on 216 longitudinal serum samples from 36 patients with advanced NSCLC receiving first-line pembrolizumab plus chemotherapy. We examined how baseline and dynamic metabolite profiles related to survival and impending disease progression, applying multivariate analyses and Random Forest (RF) modelling.

**Results:**

Lower serum levels of branched-chain amino acids (BCAAs) valine and isoleucine were associated with disease progression within 60 days. Overall survival was linked to distinct metabolomic signatures: long-term survivors showed higher serum levels of various lipids, including total phospholipids, sphingomyelin, and apolipoproteins A1 and A2. In contrast, patients who died during follow-up had higher inflammatory markers, including glycoprotein acetyls and mannose. An RF model predicted survival status with high accuracy (AUC = 0.93), with sphingomyelin, apolipoprotein A2, and glycoprotein acetyls B among the top contributors.

**Conclusion:**

Serum metabolomic profiles are closely linked to clinical outcomes in advanced NSCLC treated with pembrolizumab plus chemotherapy. Key metabolites – particularly BCAAs, lipids, and inflammatory markers – emerge as promising non-invasive biomarkers for predicting progression and survival.

## 1 Introduction

Lung cancer is the leading cause of cancer-related mortality worldwide (1). Non-small cell lung cancer (NSCLC) accounts for around 85% of cases, with most patients diagnosed at advanced stages (2). Immune checkpoint inhibitors (ICIs) targeting PD-1/PD-L1 (programmed cell death protein 1/programmed death-ligand 1) have transformed first-line management of advanced NSCLC. Pembrolizumab, an anti-PD-1 monoclonal antibody, combined with platinum-based chemotherapy, is now a standard first-line therapy for metastatic NSCLC without actionable genomic tumor aberrations (3, 4), particularly when PD-L1 tumor proportion score (TPS) is <50%. Currently, tumor PD-L1 expression is the only routinely used biomarker to guide treatment and predict response (5–7). However, accurate prediction of treatment benefit and early identification of patients at risk of rapid progression remain unmet needs.

Metabolomics has emerged as a powerful tool for identifying reliable biomarkers that capture temporal physiological changes. By profiling metabolites, this approach enables disease diagnosis, monitoring of progression, and prediction of drug response, particularly in oncology (8, 9). The rationale is that cancer and immune cells compete for nutrients, and immunosuppressive metabolic pathways can inhibit anti-tumour T cell responses (10–12). More than 150 metabolites have been associated with lung cancer in serum and tissue screens, but most studies merely compare patients to healthy controls (13). Few have assessed how serum metabolic markers correlate with clinical outcomes in advanced NSCLC (14–17). Moreover, many studies have used liquid chromatography-mass spectrometry (LC-MS) for broad small-molecule coverage. In contrast, nuclear magnetic resonance (NMR) spectroscopy provides highly reproducible absolute quantification and detailed lipoprotein subclass profiling (18, 19). These capabilities are particularly advantageous for identifying robust clinical biomarkers and tracking longitudinal changes in patient cohorts.

In this study, we performed comprehensive quantitative metabolomic profiling using NMR spectroscopy in NSCLC patients receiving pembrolizumab in combination with platinum-based chemotherapy. We aimed to determine whether serum metabolite profiles correlate with clinical outcomes, to compare short and long-term survivors, and to evaluate metabolic changes during therapy. We hypothesized that specific metabolites or metabolic pathways could serve as biomarkers of therapeutic benefit. This study – among the first comprehensive metabolomics analyses in a uniform NSCLC cohort with longitudinal follow-up – seeks to clarify how the host metabolic milieu influences immunotherapy outcomes in lung cancer.

## 2 Materials and methods

### 2.1 Study population and design

Between May 2021 and October 2023, 36 a prospective convenience sample of adult patients with histologically confirmed, advanced NSCLC Stage IV were enrolled at Technical University of Munich (TUM) University Hospital, Munich. All patients received a combination of pembrolizumab with platinum-based chemotherapy (carboplatin/pemetrexed or carboplatin/paclitaxel). Since immunotherapy is not a standard treatment for NSCLC patients with EGFR, BRAF V600E, ALK, or ROS1 alterations, individuals harboring any of these mutations or rearrangements were excluded. All patients received premedication with glucocorticoids, and folic acid plus vitamin B12 if pemetrexed was administered.

Longitudinal sample collection occurred at multiple time points approximately every 3 weeks, resulting in a total of 216 samples. The median number of samples per patient was 4 (range 1–21). Patients were instructed to refrain from eating at least 2 hours prior to blood collection. Clinical data such as age, sex, body mass index (BMI), histology, therapy line, and PD-L1 status were collected. Overall survival (OS) and progression-free survival (PFS) were calculated from initiation of chemoimmunotherapy. PD-L1 tumor proportion score (TPS) was assessed by immunohistochemistry. Radiographic response was evaluated every 8–10 weeks per RECIST v1.1. Data cut-off was January 31, 2024.

### 2.2 Baseline patient characteristics and clinical outcomes

We enrolled 36 patients with a median age of 66.5 years [range 48.0–81.6], of whom 61.1% were male (**Supplementary Table 1**). The most common histopathological subtype was adenocarcinoma (75.0%), followed by squamous cell carcinoma (16.7%). Patients with adenocarcinoma received carboplatin/pemetrexed as the chemotherapy backbone, while the remaining patients were treated with carboplatin/paclitaxel. Brain metastases were present in 38.9% of our patients at baseline. Regarding PD-L1 TPS status in our cohort, 47.2% had a TPS of <1% and 50% had a TPS of 1–49%. Median PFS was 8.8 months (95% CI 5.9 to 16.3) and median OS was 22.0 months (95% CI 14.6 to not reached) (**Supplementary Figure 1**). This aligns closely with the findings from KEYNOTE-189 for non-squamous (median PFS 11.3 months, OS 21.8 months) (3) and KEYNOTE-407 for squamous NSCLC (median PFS 8.0 months, OS 17.2 months) (4).

### 2.3 Untargeted metabolite analysis by NMR spectroscopy

Serum was obtained from whole blood by centrifugation and stored at -80 °C until NMR analysis. After thawing, 350 µl of serum was taken and mixed with 350 µl of aqueous buffer. The buffer consisted of H_2_O p.A., 0.1 g/l NaN_3_, 0.067 mol/l Na_2_HPO_4_, 0.033 mol/l Na_2_HPO_4_ (pH: 7.15 ± 0.05), 5% D_2_O as field-lock substance and an internal standard (6mM pyrazine) for quantification. From this mixture, 600 µl was transferred into a 5mm Bruker NMR tube and sealed with barcode-labelled lids. The final NMR samples were stored at refrigerator temperature after preparation until measurement for a maximum time of 4 h.

NMR measurement was performed on the spectrometer Bruker AVANCE NEO 600 MHz. Measuring time per sample was 6.5 min with the measuring method 1D 1H noesygppr1d_d20, NS = 16, T = 310 K. All measured spectra passed the quality control routine and were included in data analysis. The spectra obtained were Fourier transformed using TopSpin software (version 4.1.1 and 4.2.0, Bruker Biospin, Germany). All spectra were automatically phased and subjected to baseline correction. Subsequently, the spectra were analyzed using the proprietary lifespin Profiler software (version 1.4_Blood, version LipoPro_1.2.3_A) and a quantitative metabolite list was generated. While the NMR acquisition is untargeted, the subsequent identification and absolute quantification rely on extensive prior validation using pure standard compounds to establish exact reference spectra. Absolute quantification was achieved using pyrazine (6mM) as an internal standard. Furthermore, the assay’s robustness was previously validated via spike-in experiments in serum to account for matrix effects, and the lipoprotein subclass quantification was calibrated against ultracentrifugation and standard clinical chemistry methods (e.g., Roche-Cobas).

The NMR-based assay quantified 66 serum and 177 lipoprotein subclass parameters.

### 2.4 Multivariate model building and machine learning algorithms

Prior to multivariate model building, a data filtering step using R’s nearZeroVar function removed metabolites with minimal variance to reduce noise and improve the stability and interpretability of subsequent models. Principal Component Analysis (PCA) was employed as an unsupervised multivariate technique for initial data exploration and dimensionality reduction. PCA transformed the original dataset into a new set of orthogonal principal components (PCs) ordered such that the first few components capture the maximum possible variance, allowing for visualization of inherent data structures. Moreover, Partial Least Squares Discriminant Analysis (PLS-DA), a supervised classification method, was utilized to identify variables that maximally discriminate between predefined sample classes. PLS-DA modelled the relationship between the predictor matrix (X; metabolite data) and a categorical response variable (Y; class membership). Successive latent variables in PLS-DA were constructed to maximize the covariance between X and Y. Model performance was assessed using cross-validation, evaluating R^2^X, R^2^Y, and Q^2^Y. To further refine discrimination and enhance model interpretability, Orthogonal Partial Least Squares Discriminant Analysis (OPLS-DA) was applied. OPLS-DA separated systematic variation in X into a predictive part (correlated with Y) and an orthogonal part (uncorrelated with Y). Model validation for OPLS-DA was based on R^2^Y and Q^2^Y values.

A Random Forest (RF) algorithm was implemented for classification. Model training and performance evaluation used repeated 10-fold cross-validation with five repeats. Potential multicollinearity was assessed using variance inflation factor (VIF); highly collinear variables were considered for exclusion or handled by the inherent robustness of RF. Predictive performance was primarily evaluated using the area under the curve (AUC) of the receiver operating characteristic (ROC) curve, constructed by plotting true positive rate (sensitivity) against false positive rate (1−specificity). Metabolite importance in the RF model was quantified using the Mean Decrease Gini (MDG) index, measuring the average Gini impurity reduction per variable across trees.

### 2.5 Pathway enrichment analysis

The identified, significantly altered metabolites were subjected to pathway enrichment analysis using the FELLA package (version 1.24.0) in R (version 4.4.1), which computes node scores via a diffusion-based algorithm applied to a Kyoto Encyclopedia of Genes and Genomes (KEGG)-derived network (KeggDB Human database). Statistical significance (p-scores) for each KEGG node was computed using the normal approximation. Result tables and network plots were generated using a corrected p-score threshold of 0.10.

### 2.6 Statistical analysis

Survival curves were estimated by the Kaplan-Meier method; 95% confidence intervals (CIs) and median survival were computed via Greenwood’s formula and the Brookmeyer-Crowley method, respectively. Statistical significance in this analysis was determined using the Wilcoxon-Mann-Whitney test. Resulting p-values were corrected for multiple testing (Benjamini-Hochberg procedure); the false discovery rate (FDR) correction was applied to all parameters tested. The (un-)corrected p-values were translated into asterisk (*) notation as follows: *** indicates p < 0.001, ** indicates p < 0.01, and * indicates p<0.05. Fold change (fc) and Cohen’s d were chosen as the measures of effect size. Cohen’s d was calculated by dividing the difference of the means of the respective groups by their pooled standard deviation. All figures were generated with R (version 4.0.2, 2020).

### 2.7 Ethics

The study was conducted in accordance with the Declaration of Helsinki (as revised in 2013) and approved by the ethics committee of TUM University Hospital, Munich, Germany (code: 728/20 S-KK). Written informed consent for the metabolome profiling was obtained from all participants.

## 3 Results

### 3.1 Influence of sex on the serum metabolome

As expected, several sex-related differences were evident in the serum metabolome of our NSCLC cohort (**Supplementary Figure 2**). PLS-DA showed moderate discrimination (R²Y=0.638, Q²Y=0.581), with lipoprotein and amino acid parameters contributing most heavily to sex separation – mainly high-density lipoprotein (HDL) cholesterol and apolipoprotein A1 and A2. After FDR correction, 31 metabolites/lipoprotein parameters differed significantly between sexes (**Supplementary Table 2**). Male samples exhibited lower mean HDL cholesterol (fc=0.66, d=-1.82), apolipoprotein A1 (fc=0.74, d=-1.59), and apolipoprotein A2 (fc=0.77, d=–1.45) relative to females. Conversely, males had higher mean total triglycerides (fc=1.47, d=0.77), leucine (fc=1.21, d=0.79), and glucose (fc=1.27, d=0.67) compared to females.

### 3.2 Metabolic prediction of impending disease progression

To investigate whether the metabolome could forecast disease progression prior to radiological confirmation, we compared metabolic profiles from samples taken up to 60 days before confirmed progressive disease (PD) (N = 25 samples from 15 patients; ‘PD’ group) with samples from patients who never showed progression (N = 48 samples from 10 unique patients; ‘no PD’ group). Both groups consisted of 60% male patients.

**Figure 1:**
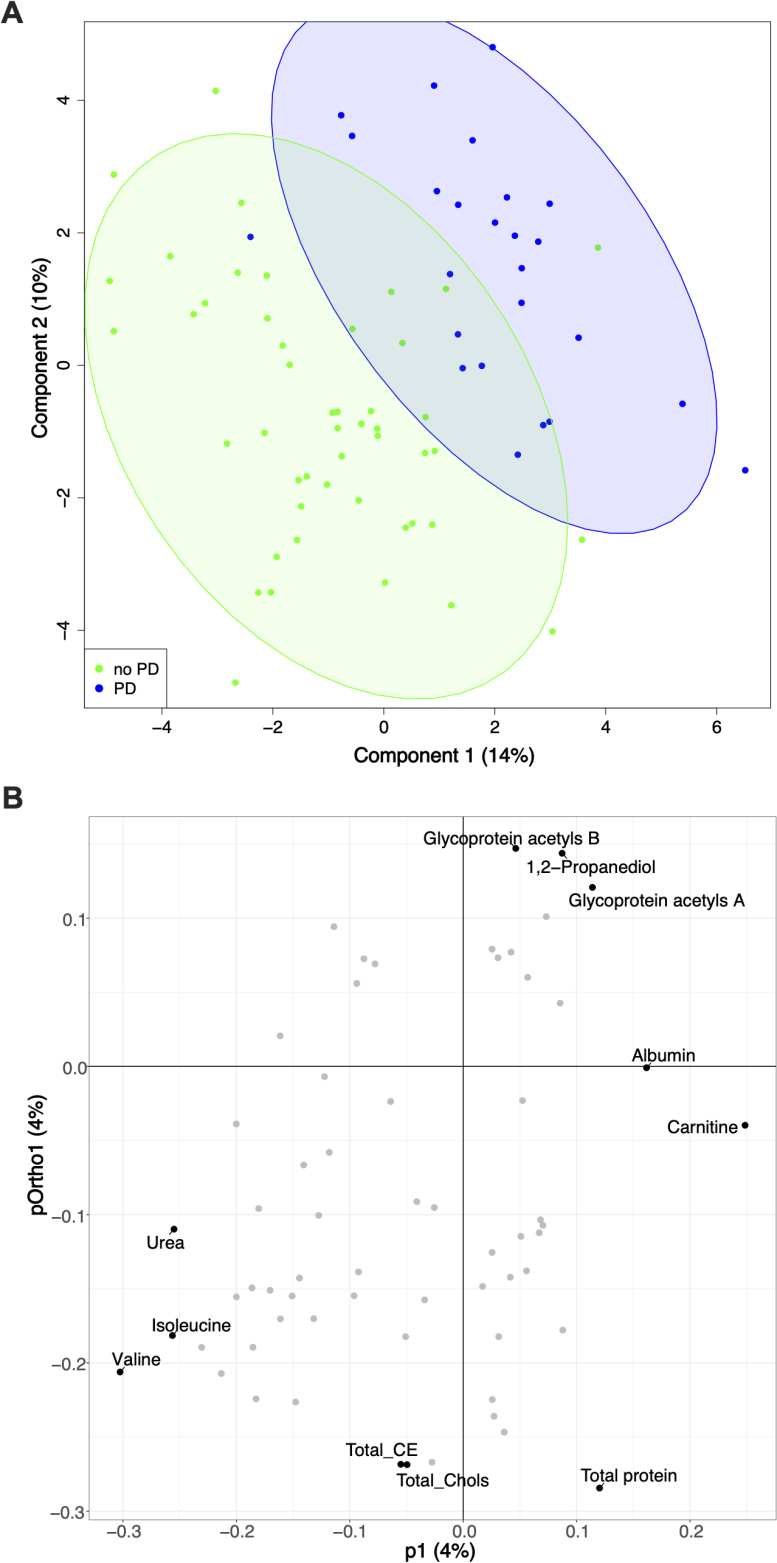
Metabolic discrimination of progressive disease (PD). **(A)** PLS-DA scores showing separation of patients with ‘PD’ (blue) from those without (‘no PD’, green). **(B)** Corresponding OPLS-DA loadings plot highlighting key metabolites driving separation PD, progressive disease; CE, cholesterol ester; Chols, cholesterol.

Multivariate analysis was employed to assess overall metabolic differences. PLS-DA scores plot indicated a degree of separation between the ‘no PD’ group (green) and the ‘PD’ group (blue) based on their overall metabolic profiles (**Figure 1A**). Further, the OPLS-DA model effectively discriminated between the groups, demonstrating robust performance with an R^2^Y of 0.813 and a Q^2^Y of 0.551. The OPLS-DA loadings plot shows the metabolites contributing most to this separation – such as valine, isoleucine, total protein, and glycoprotein acetyls A (**Figure 1B**).

Univariate analysis correspondingly identified specific metabolites differing significantly between patients who progressed within 60 days and those who did not (**Table 1**). After correction for multiple testing, only three metabolites remained statistically significant. Notably, lower levels of the branched-chain amino acids (BCAAs) valine (fc=0.79, d=-1.04) and isoleucine (fc=0.80, d=-0.75) were characteristic of the ‘PD’ group. Acetoacetic acid was also notably lower in the ‘PD’ group (fc=0.60), but its smaller effect size (d=-0.31) indicates a weaker and less robust difference than for valine and isoleucine.

**Table 1:** Statistically significant metabolites – progression within 60 days versus no progression.

Metabolite	p-value	p-value	p-value (corr.)	p-value (corr.)	Fold change^†^	Cohen’s d
Valine	<0.001	***	0.01	*	0.79	-1.04
Acetoacetic acid	0.001	**	0.03	*	0.60	-0.31
Isoleucine	0.001	**	0.03	*	0.80	-0.75
Histidine	0.01	**	0.11	n.s.	0.84	-0.68
Leucine	0.01	*	0.11	n.s.	0.84	-0.70
Urea	0.01	*	0.11	n.s.	0.72	-0.74
Carnitine	0.01	*	0.11	n.s.	1.33	0.78
Lysine	0.02	*	0.14	n.s.	0.84	-0.64
Phenylalanine	0.02	*	0.14	n.s.	0.75	-0.59
Tyrosine	0.02	*	0.15	n.s.	0.83	-0.65
Creatine	0.03	*	0.15	n.s.	0.69	-0.51
Ornithine	0.03	*	0.15	n.s.	0.83	-0.53
Albumin	0.03	*	0.15	n.s.	1.06	0.53
Methanol	0.03	*	0.15	n.s.	0.81	-0.46
HDL cholesterol	0.03	*	0.21	n.s.	0.87	-0.53
Apolipoprotein A1	0.04	*	0.21	n.s.	0.89	-0.52

corr., corrected; HDL, high density lipoprotein; n.s., not significant; *** p-value <0.001; ** p-value <0.01; * p-value <0.05. ^†^ progression (‘PD’) over no progression (‘no PD’).

**Figure 2:**
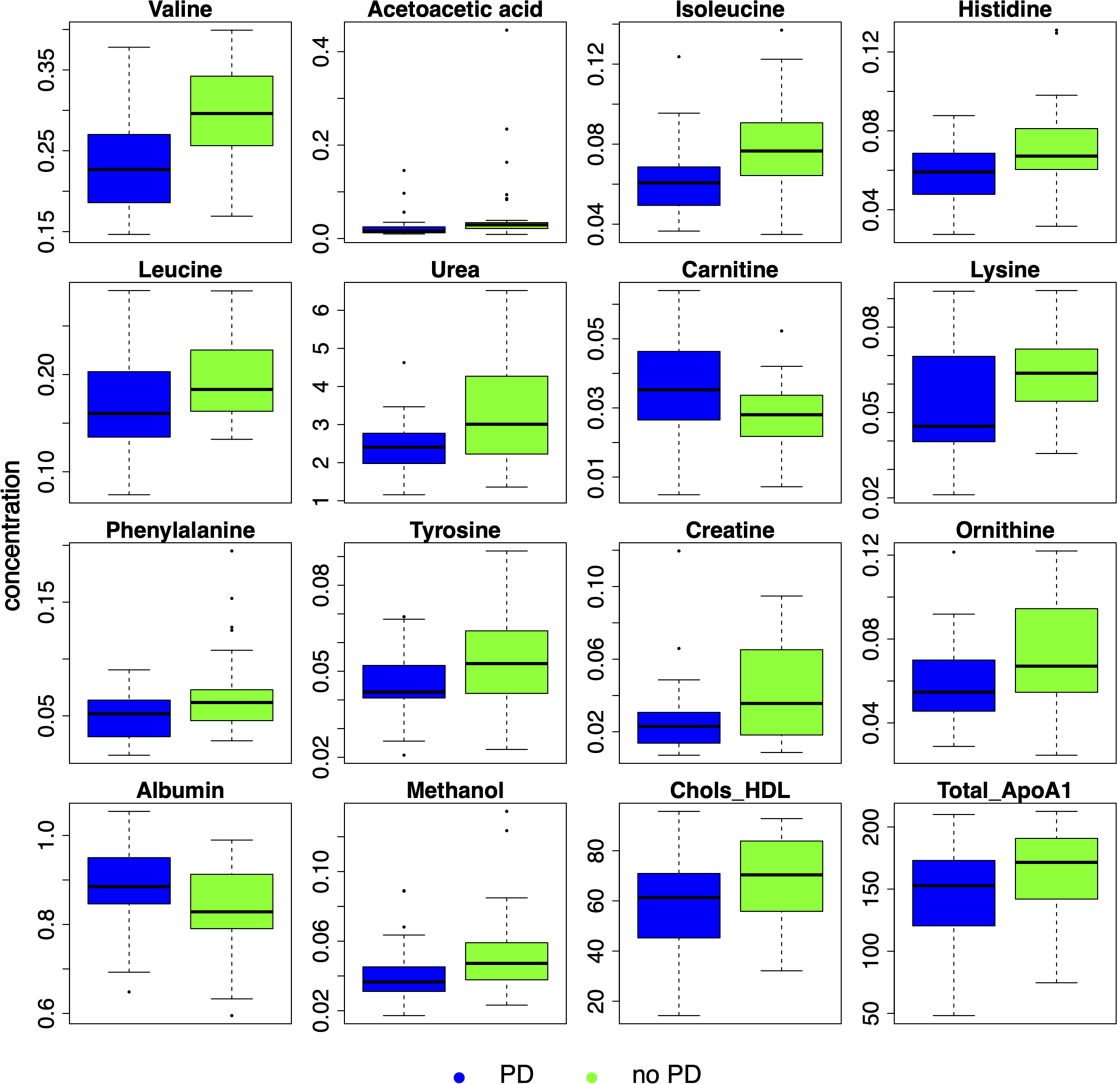
Detailed analysis of selected metabolites discriminating progressive disease (PD). Box plots comparing concentrations of selected metabolites between PD (blue) and no PD (green) groups. Chols_HDL, high-density lipoprotein cholesterol; ApoA1, apolipoprotein A1.

The concentration differences for these and other selected metabolites are illustrated in the box plots (**Figure 2**).

These findings suggest that reduced serum concentrations of the BCAAs valine and isoleucine could potentially serve as early indicators of impending disease progression.

### 3.3 Metabolic signatures associated with overall survival

Complementing the analysis of disease progression, we assessed the metabolomic signature’s ability to predict overall survival. To this end, we analyzed metabolic profiles from patients who died during follow-up (survival ‘no’ group with 96 samples from 22 unique patients, 61% male) and patients still living at study end (survival ‘yes’ group with 120 samples from 14 unique patients, 64% male). Initial multivariate analysis using PLS-DA demonstrated a separation between the surviving patients (‘yes’, red) and the deceased patients (‘no’, blue) based on their overall metabolic profiles (**Supplementary Figure 3A**), suggesting distinct metabolic signatures associated with outcome.

Univariate analysis revealed numerous metabolites differing significantly between the two groups, with 29 of these (listed in **Table 2**) remaining statistically significant after correction for multiple testing. Notably, total phospholipids (fc=1.20, d=0.89), sphingomyelin (fc=1.31, d=0.39), and phosphatidylcholine (fc=1.20, d=0.81) were among the most significantly elevated metabolites in the survival group compared to the deceased group. Other altered metabolites included members of the lipid transport system, such as apolipoprotein A1 (fc=1.20, d=0.81), apolipoprotein A2 (fc=1.20, d=0.88), and various cholesterol fractions (total cholesterol, total cholesterol ester, total free cholesterol, and HDL cholesterol), all of which were generally higher in the survival group. Conversely, inflammatory metabolites such as glycoprotein acetyls A (fc=0.90, d=-0.52), glycoprotein acetyls B (fc=0.89, d=-0.50), as well as mannose (fc=0.82, d=-0.53), and 3-hydroxybutyric acid (fc=0.44, d=-0.31) were significantly higher in the deceased group. Box plots visually illustrate the concentration distributions for a selection of these key differentiating metabolites (**Supplementary Figure 3B**).

**Table 2:** Statistically significant metabolites – survival versus deceased.

Metabolite	p-value (corr.)	p-value (corr.)	Fold change^‡^	Cohen’s d
Phospholipids (total)	<0.001	***	1.20	0.89
Apolipoprotein A2	<0.001	***	1.20	0.88
Sphingomyelin	<0.001	***	1.31	0.39
Apolipoprotein A1	<0.001	***	1.20	0.81
Phosphatidylcholine	<0.001	***	1.20	0.81
Cholesterol (total)	<0.001	***	1.19	0.80
Cholesterol ester (total)	<0.001	***	1.19	0.80
Free cholesterol (total)	<0.001	***	1.19	0.75
Ornithine	<0.001	***	1.24	0.67
Triglycerides (total)	<0.001	***	1.25	0.44
Glycoprotein acetyls A	<0.001	***	0.90	-0.52
HDL cholesterol	<0.001	***	1.21	0.61
myo-Inositol	<0.001	***	1.87	0.58
Mannose	<0.001	***	0.82	-0.53
Protein (total)	<0.001	***	1.05	0.59
Apolipoprotein B	<0.001	***	1.18	0.54
Glycoprotein acetyls B	<0.001	***	0.89	-0.50
LDL cholesterol	0.004	**	1.18	0.51
Formic acid	0.004	**	0.82	-0.38
Serine	0.004	**	1.13	0.45
Alanine	0.004	**	1.18	0.48
3-Hydroxybutyric acid	0.005	**	0.44	-0.31
Proline	0.007	**	1.14	0.36
Dimethylsulfone	0.009	**	0.84	-0.4
Methanol	0.01	*	1.14	0.28
Glycine	0.02	*	1.10	0.4
Lysine	0.02	*	1.08	0.25
1,2-Propanediol	0.03	*	0.18	-0.30
Tyrosine	0.04	*	1.07	0.21

corr., corrected; HDL, high density lipoprotein; LDL, low density lipoprotein; *** p-value <0.001; ** p-value <0.01; * p-value <0.05. ^‡^ survived (‘yes’) over deceased (‘no’).

To further elucidate the biological significance of these alterations, we performed a diffusion-based KEGG pathway enrichment analysis. This analysis revealed that the metabolic networks most significantly impacted between survival groups were primarily associated with lipid dynamics (e.g., cholesterol metabolism and glycosphingolipid biosynthesis), as well as critical immune and cellular responses, including antigen processing and presentation, and apoptosis (**Supplementary Figure 4**).

**Figure 3:**
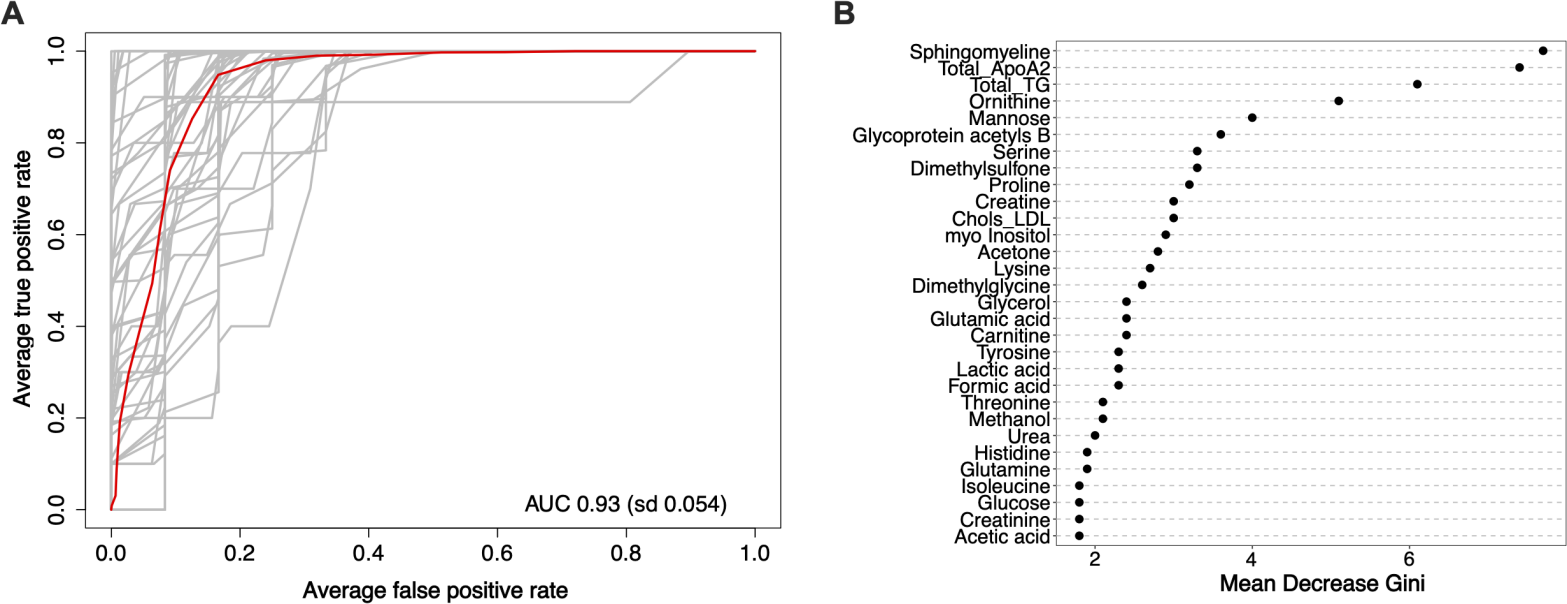
Machine learning analyses discriminating between survival groups based on metabolic profiles. **(A)** Receiver Operating Characteristic (ROC) curve from a Random Forest classification model, evaluating the model’s performance in distinguishing between the survival ‘yes’ and ‘no’ groups. **(B)** Metabolite importance plot derived from the Random Forest model, ranking metabolites by their Mean Decrease Gini score. AUC, area under the curve; ApoA1, apolipoprotein A1; TG, triglycerides; Chols_LDL, low-density lipoprotein cholesterol.

Next, to assess the predictive capability of the metabolic signature, an RF classification model was developed. The model demonstrated excellent performance in distinguishing between the survival and deceased groups, achieving an average AUC-ROC of 0.93 (standard deviation=0.054) (**Figure 3A**). The metabolite importance plot derived from this RF model highlighted sphingomyelin, apolipoprotein A2, total triglycerides, ornithine, mannose, and glycoprotein acetyls B as some of the most influential metabolites contributing to the model’s discriminatory power (**Figure 3B**). A detailed breakdown of this classification performance is provided in a confusion matrix (**Supplementary Figure 5**), illustrating the model’s robustness with minimal false positive (3.6%) and false negative (7.5%) misclassifications.

### 3.4 Longitudinal metabolic dynamics during treatment and association with survival

**Figure 4:**
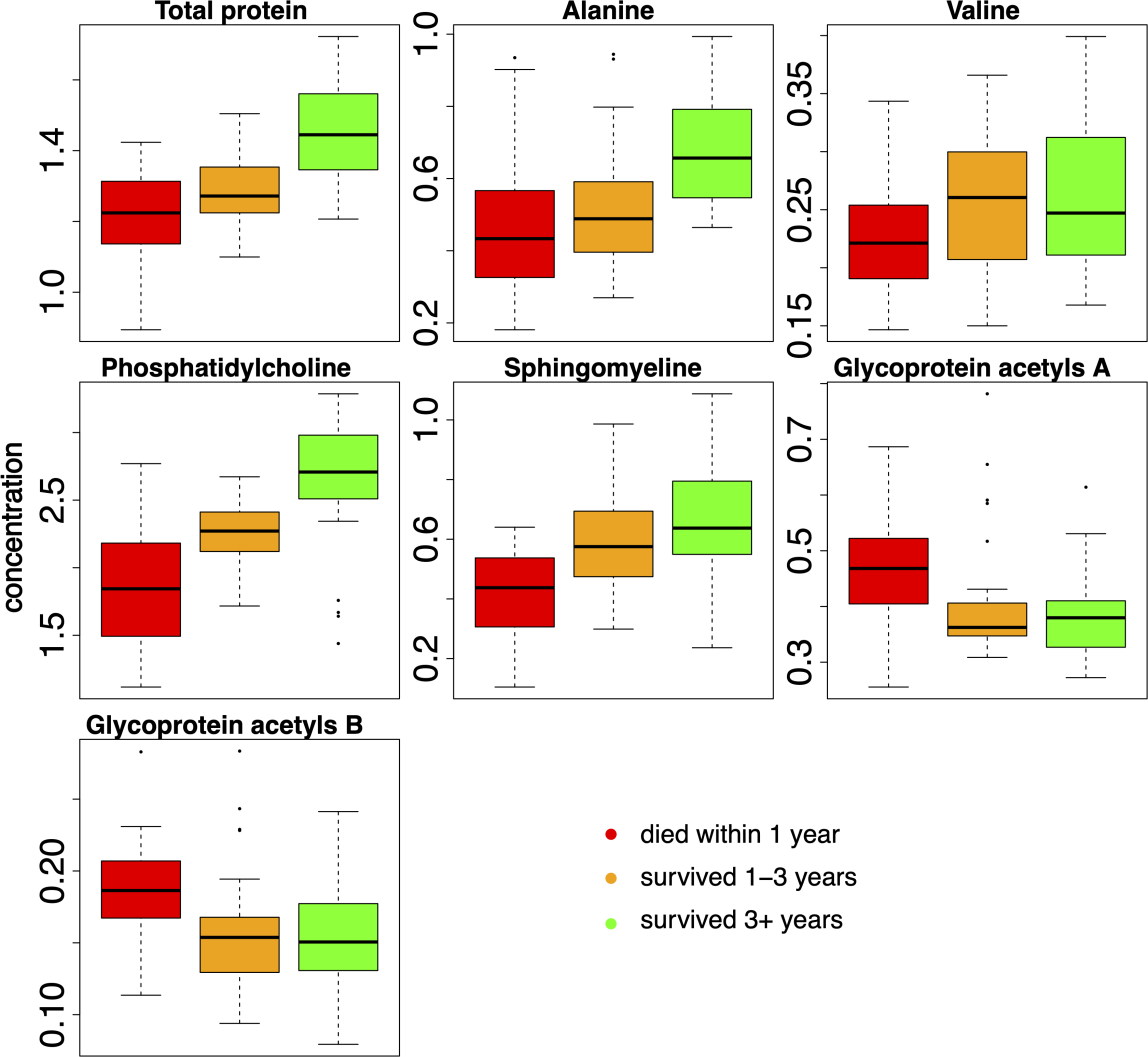
Comparison of serum metabolite levels across patient groups defined by overall survival duration. Box plots showing the concentration of selected key metabolites in [mmol/L] or [mg/dL] for patients who died within <1 year (red), survived 1–3 years (orange), and survived >3 years (green). Samples for survivor groups were restricted to the corresponding time periods. Box plots depict median and interquartile ranges.

To further explore the relationship between metabolic profiles and long-term outcomes, we stratified patients into three groups based on their overall survival duration: those who died within 1 year (‘<1 year’, red), those who survived between 1 and 3 years (‘1–3 years’, orange), and long-term survivors living beyond 3 years (‘>3 years’, green) (**Figure 4**).

Metabolite concentrations showed distinct patterns across survival groups. Regarding markers related to protein metabolism and amino acid availability, serum levels of total protein, alanine, and valine generally showed a positive association with survival duration. Patients who survived >3 years (green) tended to exhibit the highest median concentrations of these metabolites, reflecting potentially better nutritional status or preserved protein synthesis, while those who died within 1 year (red) displayed the lowest. A similar positive trend with survival duration was observed for key lipid components integral to cell membrane structure and signaling pathways. Phosphatidylcholine and sphingomyelin concentrations were generally highest in the long-term survivors (‘>3 years’, green) and lowest in patients with the poorest survival (‘<1 year’, red).

In contrast, serum levels of the inflammatory markers glycoprotein acetyls A and B exhibited an inverse relationship with survival. Patients who died within 1 year (green) tended to have the highest median concentrations of these glycoproteins, suggesting a higher systemic inflammatory state. Interestingly, the 1–3-year survival group (orange) and the >3-year survivors (green) generally displayed markedly lower median levels of these inflammatory markers compared to the short-term survival group.

Longitudinal analysis of metabolic dynamics within the first 250 days after initial sample collection revealed distinct trajectories between survivors and non-survivors. Of note, glycoprotein acetyls A and B levels at first draw were significantly higher in patients who died during our study (survival ‘no’) and generally showed an increasing trend over time (**Supplementary Figure 6**). Furthermore, survivors, particularly males, often showed stable or increasing trends for various cholesterol species (e.g., total cholesterol, low-density lipoprotein [LDL] cholesterol) and apolipoprotein B, whereas non-survivors exhibited declining levels of these markers. Collectively, these longitudinal metabolic shifts suggest that longitudinal monitoring may provide earlier prognostic information than single baseline measurements.

## 4 Discussion

This study demonstrates that serum metabolomic profiles in advanced NSCLC patients receiving first-line pembrolizumab plus platinum-based chemotherapy are significantly associated with imminent disease progression and overall survival. Our findings in this cohort support the growing understanding that the host’s systemic metabolic state is a critical determinant and predictor of immunotherapy efficacy, extending prior work on metabolomics in lung cancer (13–15).

### 4.1 Pre-progression signature

Notably, we identified a distinct metabolic signature preceding radiographic disease progression. Patients who experienced progressive disease within 60 days of sample collection had significantly lower levels of BCAAs valine and isoleucine compared to those who did not progress. Reduced circulating BCAAs have previously been linked to cancer cachexia and poorer overall survival (20), and recent metabolomic studies have identified BCAAs signatures predictive of response to PD-1 blockade in NSCLC (21). Our data suggest that lower levels of these essential amino acids might precede radiologically detectable progression, potentially reflecting altered tumor or host amino acid metabolism. One hypothesis is that rapidly proliferating tumors consume BCAAs or that early cachexia lowers circulating BCAAs before radiological changes become evident. Decreased BCAAs could also indicate upregulated catabolic pathways in T cells, linking BCAAs to immunosuppression.

### 4.2 Survival signatures

Furthermore, we identified distinct metabolic signatures associated with overall survival, thereby identifying patients with a better prognosis. Our RF model predicted survival status with high accuracy (AUC 0.93), with sphingomyelin, apolipoprotein A2, total triglycerides, ornithine, mannose, and glycoprotein acetyls B being key contributors. The importance of ornithine, a key metabolite in the urea cycle and arginine metabolism, is particularly notable given the role of arginine in T-cell function and its depletion in the tumor microenvironment by enzymes such as arginase (22–24).

Long-term survivors exhibited significantly higher levels of several lipid species, notably major phospholipids such as phosphatidylcholine and sphingomyelin, alongside apolipoproteins and various cholesterol fractions. The elevated levels of key phospholipids, particularly sphingomyelin, align with findings that increased serum sphingolipid concentrations positively correlate with OS in NSCLC patients (25–27). Sphingolipid metabolism, integral to membrane stability and signaling, is known to be among the most dysregulated processes in NSCLC (28). Indeed, these lipids have been implicated in modulating tumor-immune interactions and T-cell responses (29–31). Tumors also directly modify their lipid membrane to facilitate invasion and promote lipid metabolism for energy and protection from oxidative stress and to form early metastases (17, 32).

Conversely, patients with shorter survival had higher serum levels of glycoprotein acetyls A and B, mannose, and ketone bodies like 3-hydroxybutyric acid. Elevated ketone bodies are a hallmark of cancer-associated cachexia, and high circulating mannose and mannose-rich glycan signatures have been associated with lymph-node metastasis, advanced stage and poorer prognosis in colorectal and breast cancer (33–35). More importantly, high levels of glycoprotein acetyls are established markers of systemic inflammation and have been linked to increased mortality and adverse events in various chronic inflammatory conditions and cancer (36–39). Large prospective metabolomics studies also relate higher circulating glycoprotein acetyls-like inflammatory signals to increased lung cancer risk (40).

### 4.3 Contextualization

Our findings align with and expand upon recent metabolomic trials in advanced NSCLC by confirming the prognostic value of specific metabolite classes. Regarding lipids, our observation that elevated phospholipids and sphingomyelin correlate with prolonged survival is consistent with prior studies, which linked baseline phosphatidylcholines and sphingolipids to favorable platinum-based chemotherapy responses (41, 42). Furthermore, our finding that reduced BCAAs precede early progression complements recent chemoimmunotherapy studies (16, 43). While those studies identified other specific amino acid derivatives such as N-(3-Indolylacetyl)-L-alanine and tryptophan metabolites as independent predictors of PFS, our data specifically highlight the systemic depletion of essential BCAAs as an early indicator of treatment failure. Finally, while our study highlights glycoprotein acetyls as strong indicators of systemic inflammation and poor survival, similar inflammatory and energy-related metabolic shifts have been shown to predict poor immunotherapy outcomes (15).

### 4.4 Limitations

This study has several limitations. First, the modest sample size (N = 36) reduced statistical power, particularly for subgroup analyses, and the single-center design may limit generalizability. External validation in larger, independent cohorts will be essential to confirm these findings. Second, our primary outcome analyses were not stratified by sex because of limited sample size. In our cohort, females had higher mean levels of HDL cholesterol and its associated apolipoproteins, whereas males had higher triglycerides, leucine, and glucose. While subgroups showed highly similar sex distributions, with 60–65% male patients, future metabolomic studies should aim to stratify analyses by sex, particularly given known sex differences in immune responses and ICI outcomes (9, 44, 45). Third, unmeasured confounders such as detailed dietary intake and specific comorbidities could have influenced serum metabolite levels. Finally, the NMR-based metabolomic platform, while comprehensive for lipids and selected small molecules, may not capture all relevant metabolic alterations. In addition, emerging data indicate that the gut microbiome and microbiome-derived metabolites modulate immunotherapy efficacy in lung cancer, which we were unable to assess in this study (46, 47).

### 4.5 Implications

Despite these limitations, our findings have important translational implications. The identified metabolic signatures, particularly the robust prediction of survival and the early indication of progression by BCAAs, suggest that serum metabolomics could complement existing biomarkers like PD-L1 expression. For instance, assessing baseline lipid profiles or BCAA levels could help stratify patients for risk or potentially guide supportive interventions. Metabolic interventions such as BCAA supplementation warrant exploration in early clinical trials. The differing longitudinal trajectories of inflammatory markers (glycoprotein acetyls A and B) and potentially other lipids further suggest that on-treatment metabolic monitoring could provide early warnings of treatment resistance and adverse outcomes. Other RF-based metabolomic models have achieved similarly high discriminative performance for chemoimmunotherapy outcomes in advanced NSCLC, supporting the potential robustness of our approach (16, 48). Finally, the minimally invasive and easily repeatable nature of serum-based assays supports their feasibility for routine clinical implementation.

### 4.6 Future work

As our data are hypothesis-generating, external validation in larger, independent, multi-center cohorts is required to confirm these findings and establish the true clinical utility of the predictive model. Subsequent studies should also explore and compare alternative machine learning algorithms, such as Support Vector Machines (SVM) or neural networks, to further optimize predictive performance and validate the stability of the identified metabolic signatures. Furthermore, the exact mechanistic roles of these altered metabolites, such as how systemic BCAA depletion or elevated glycoprotein acetyls directly influence T-cell exhaustion or tumor progression, warrant further in-depth investigation. Moreover, future functional studies using *in vitro* co-culture models and *in vivo* isotope tracing are necessary to determine whether these metabolites are merely byproducts of disease progression or active drivers of immune resistance.

## 5 Conclusion

In conclusion, this study provides hypothesis-generating evidence that serum metabolomics yields prognostically relevant information in advanced NSCLC patients receiving pembrolizumab plus platinum-based chemotherapy. We identified distinct metabolic profiles and dynamic on-treatment changes associated with overall survival and disease progression. These findings underscore the intricate link between host metabolism and response to chemoimmunotherapy and highlight the potential of metabolomic biomarkers to refine prognostication and personalize treatment strategies in NSCLC. Further research integrating metabolomics with other multi-omic data and detailed clinical information is warranted to fully realize its potential in advancing precision immuno-oncology.

## Data Availability

The raw data supporting the conclusions of this article will be made available by the authors, without undue reservation.
