# Supplementary material for: Serum metabolomic signatures predict clinical outcomes in advanced non-small cell lung cancer treated with pembrolizumab plus platinum-based chemotherapy
Source: Front Immunol. 2026 May 18;17:1770764. doi: 10.3389/fimmu.2026.1770764 (PMC13223046; doi:10.3389/fimmu.2026.1770764)
Supplement: Supplementary file 1 [file Table1.docx]

**Supplement**

**Supplementary Table S1: Patient demographics and baseline clinical characteristics (N=36).** TPS=tumour proportion score; n.a.=not available; BMI=body mass index.

|  |  | N=36 | (100.0%) |
| --- | --- | --- | --- |
| **Sex** |  |  |  |
|  | Male | 22 | (61.1) |
|  | Female | 14 | (38.9) |
| **Age (years)** | |  |  |
|  | Median [range] | 66.5 [48.0-81.6] |  |
|  | <65 | 15 | (41.7) |
|  | ≥65 | 21 | (58.3) |
| **Histology** | |  |  |
|  | Adenocarcinoma | 27 | (75.0) |
|  | Squamous cell carcinoma | 6 | (16.7) |
|  | Other | 3 | (8.3) |
| **Chemotherapy** | |  |  |
|  | Carboplatin/Pemetrexed | 27 | (75.0) |
|  | Carboplatin/Paclitaxel | 9 | (25.0) |
| **Site of metastasis** | |  |  |
|  | Brain | 14 | (38.9) |
|  | Bone | 15 | (41.7) |
|  | Adrenal | 10 | (27.8) |
|  | Liver | 6 | (16.7) |
| **PD-L1 (TPS)** | |  |  |
|  | <1% | 17 | (47.2) |
|  | 1–49% | 18 | (50.0) |
|  | ≥50% | 0 | (0.0) |
|  | n.a. | 1 | (2.8) |
| **BMI (kg/m^2^)** | |  |  |
|  | Median [range] | 22.7 [16.1-37.7] |  |
|  | <20 | 8 | (22.2) |
|  | 20–25 | 19 | (52.8) |
|  | >25 | 9 | (25.0) |
| **Number of visits** | |  |  |
|  | Median [range] | 4 [1-21] |  |

**Supplementary Table S2: Statistically significant metabolites – male versus female.** corr.=corrected; HDL=high density lipoprotein; n.d.=not defined; *** p-value <0.001; ** p-value <0.01; * p-value <0.05. ^§^ male over female.

| **Metabolite** | **p-value (corr.)** |  | **Fold change**^§^ | **Cohen's d** |
| --- | --- | --- | --- | --- |
| HDL cholesterol | <0.001 | *** | 0.66 | -1.82 |
| Apolipoprotein A1 | <0.001 | *** | 0.74 | -1.59 |
| Apolipoprotein A2 | <0.001 | *** | 0.77 | -1.45 |
| Phospholipids (total) | <0.001 | *** | 0.80 | -1.25 |
| Creatine | <0.001 | *** | 0.59 | -1.09 |
| Phosphatidylcholine | <0.001 | *** | 0.79 | -1.22 |
| Dimethylglycine | <0.001 | *** | 1.67 | 0.48 |
| Triglycerides (total) | <0.001 | *** | 1.47 | 0.77 |
| Leucine | <0.001 | *** | 1.21 | 0.79 |
| Sphingomyelin | <0.001 | *** | 1.44 | 0.51 |
| Glucose | <0.001 | *** | 1.27 | 0.67 |
| Isoleucine | <0.001 | *** | 1.22 | 0.68 |
| Lactic acid | <0.001 | *** | 1.27 | 0.62 |
| Choline | <0.001 | *** | 0.76 | -0.55 |
| Alanine | <0.001 | *** | 1.22 | 0.56 |
| 3-Hydroxybutyric acid | <0.001 | *** | 0.69 | -0.15 |
| Mannose | <0.001 | *** | 1.20 | 0.47 |
| Glutamic acid | 0.001 | ** | 1.24 | 0.51 |
| 2-Aminobutyric acid | 0.002 | ** | 0.64 | -0.48 |
| Methionine | 0.002 | ** | 3.79 | 0.47 |
| Phenylalanine | 0.003 | ** | 1.28 | 0.47 |
| Creatinine | 0.004 | ** | 1.15 | 0.41 |
| Glycine | 0.007 | ** | 0.89 | -0.53 |
| Sarcosine | 0.008 | ** | n.d. | 0.40 |
| Proline | 0.009 | ** | 1.17 | 0.43 |
| Glycerol | 0.01 | * | 1.52 | 0.46 |
| Carnitine | 0.01 | * | 1.20 | 0.46 |
| Ascorbic acid | 0.01 | * | 0.48 | -0.43 |
| Pyruvic acid | 0.01 | * | 1.28 | 0.47 |
| Acetone | 0.02 | * | 0.95 | -0.06 |
| Cholesterol ester (total) | 0.049 | * | 0.91 | -0.43 |


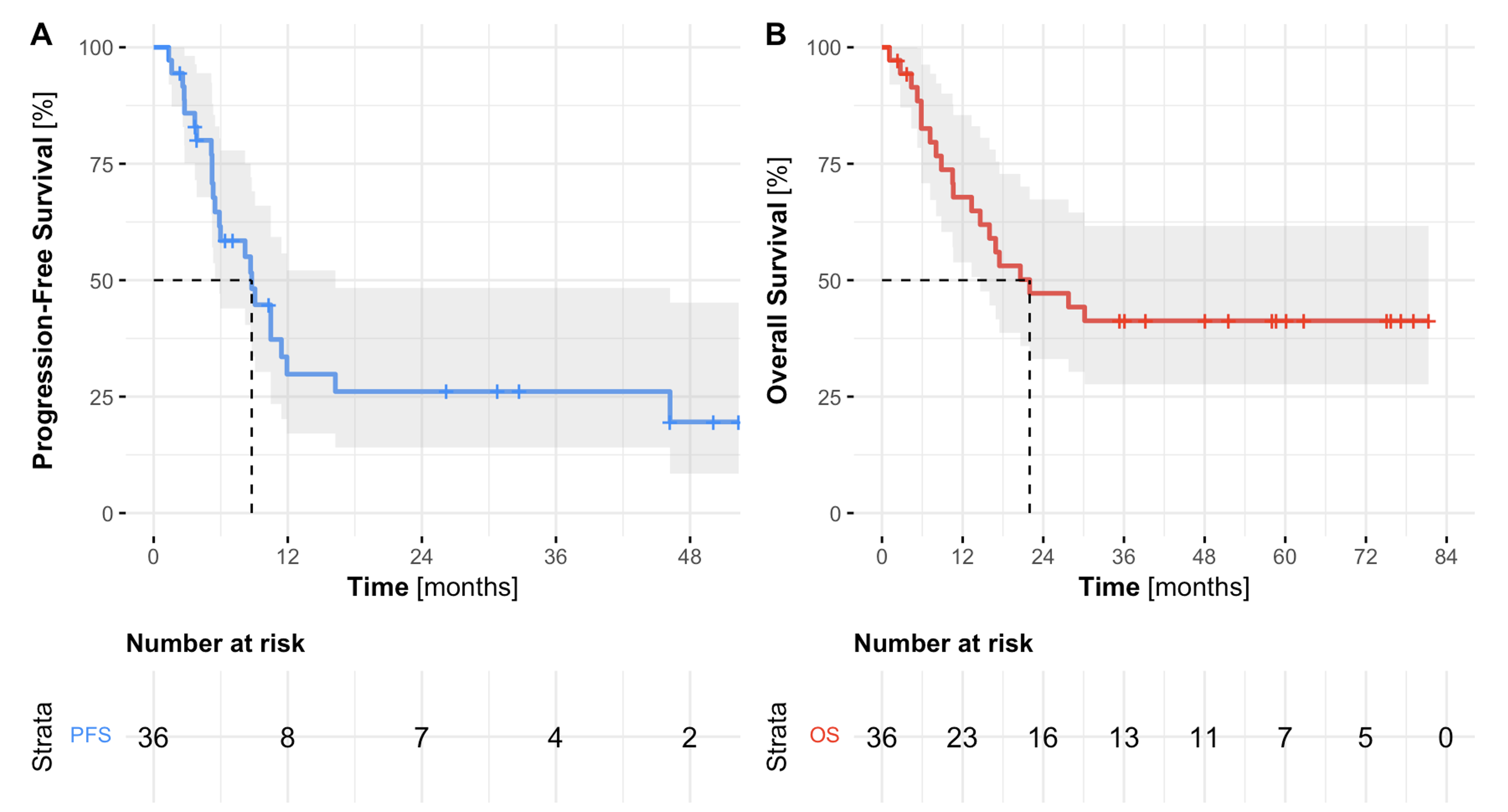


**Supplementary Figure S1: Kaplan-Meier survival curves.** (A) Progression-Free Survival (PFS) and (B) Overall Survival (OS) in our cohort. Shaded areas indicate 95% confidence intervals, dotted lines correspond to median survival, and tick marks represent censored events.


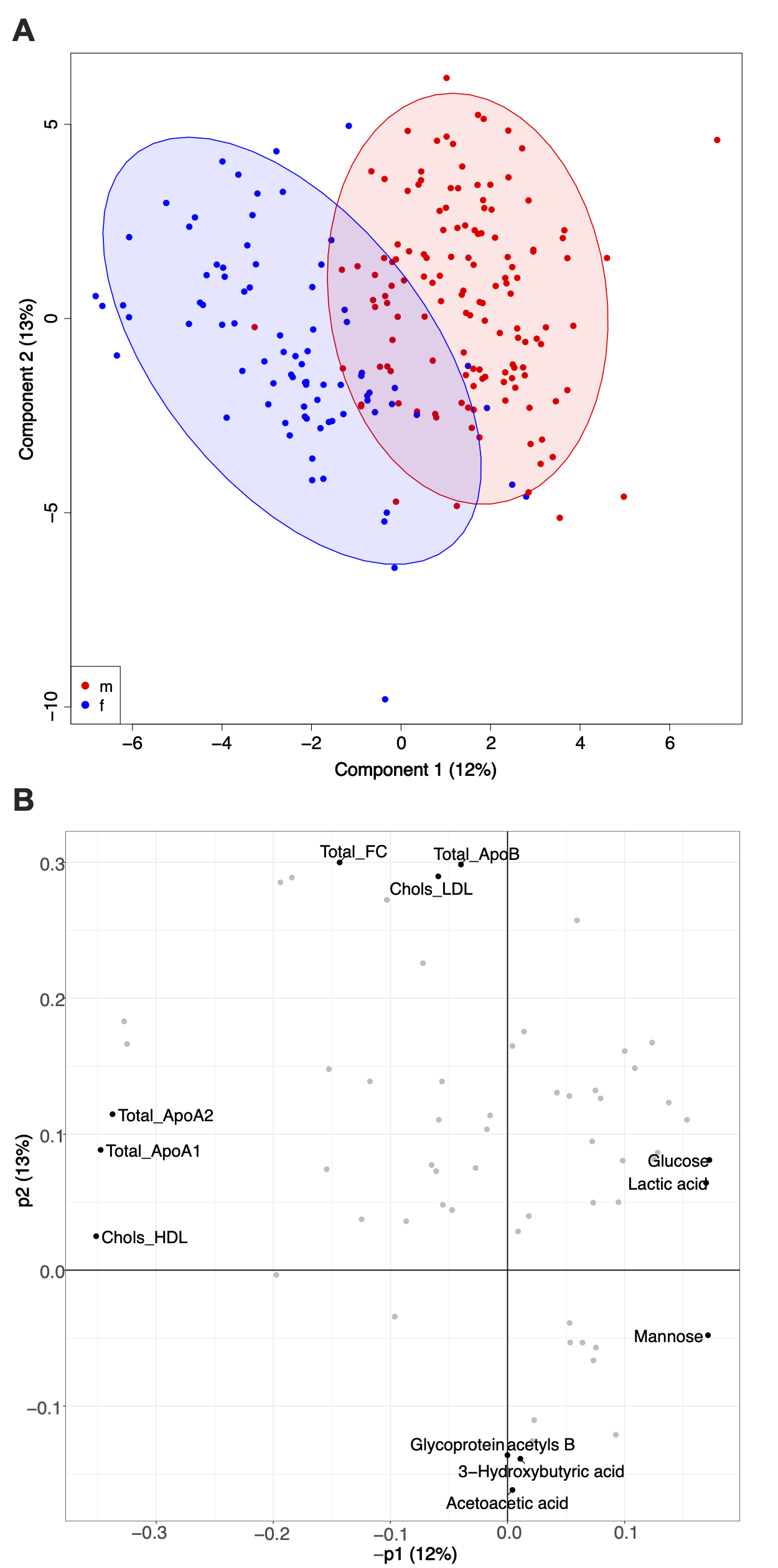


**Supplementary Figure S2:** **Metabolic profiling distinguishes male and female subjects.** **(A)** PLS-DA scores plot depicting male ('m', red) and female ('f', blue) sample clusters with 95% confidence ellipses. **(B)** PLS-DA loadings plot identifying key discriminatory metabolites. Important metabolites are labelled. m=male; f=female; Chols_HDL=high density lipoprotein cholesterol, ApoA1/ApoA2/ApoB=apolipoprotein A1/A2/B; FC=free cholesterol; Chols_LDL=low density lipoprotein cholesterol.


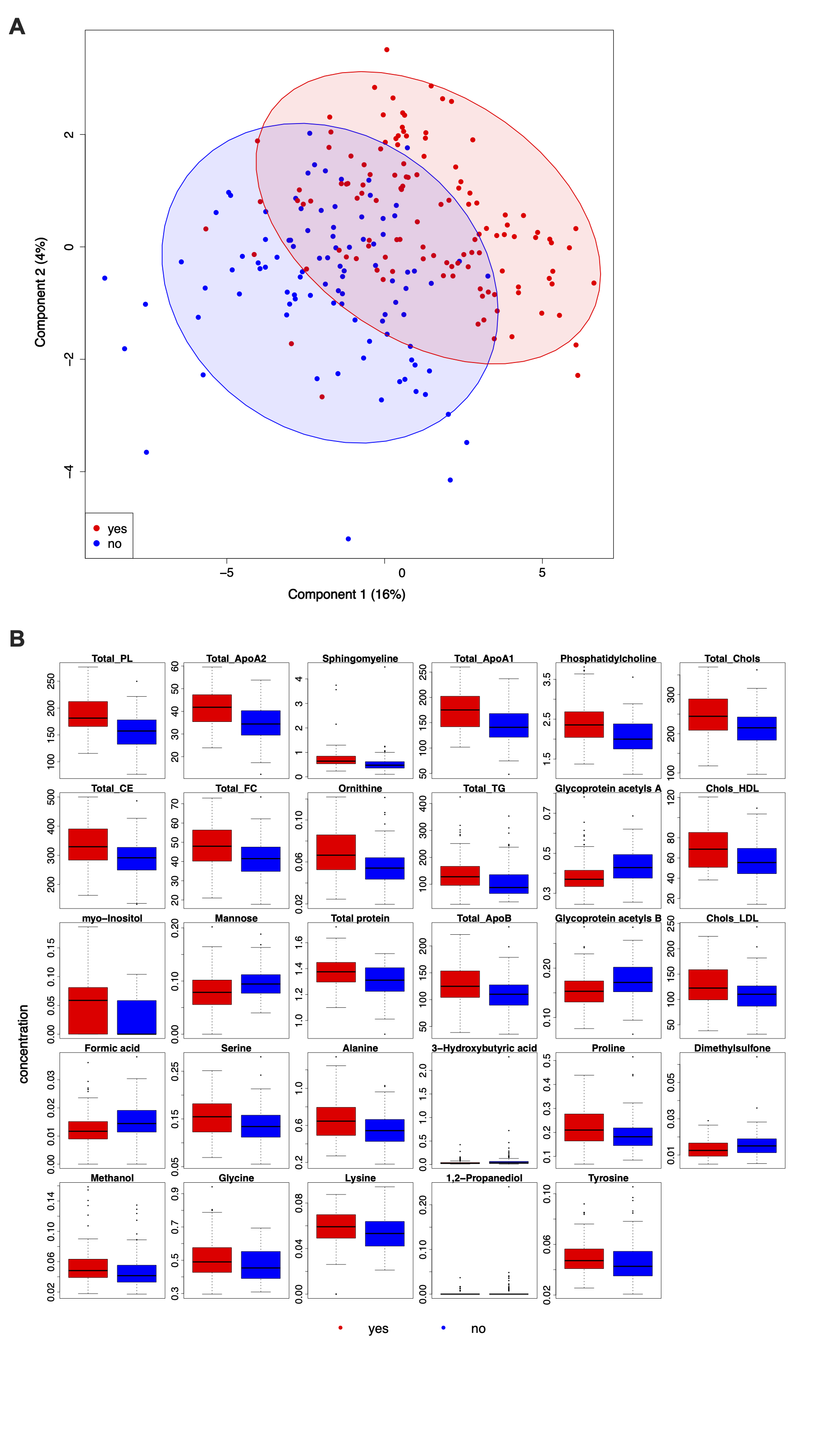


**Supplementary Figure S3: Differential metabolic profiles between survival groups based. (A)** PLS-DA scores plot illustrating the separation between subjects in the survival group ('yes', red) and the deceased group ('no', blue) based on their overall metabolic profiles. Ellipses represent 95% confidence regions for each group. **(B)** Box plots showing the concentration of selected key metabolites in [mmol/L] or [mg/dL]. Box plots depict median and interquartile ranges. PLS-DA=partial least squares-discriminant analysis; PL=phospholipids; ApoA1/A2/B=apolipoprotein A1/A2/B; Chols=cholesterol; CE=cholesterol ester; FC=free cholesterol; TG=triglycerides; Chols_HDL=high density lipoprotein cholesterol; Chols_LDL=low density lipoprotein LDL cholesterol.

**
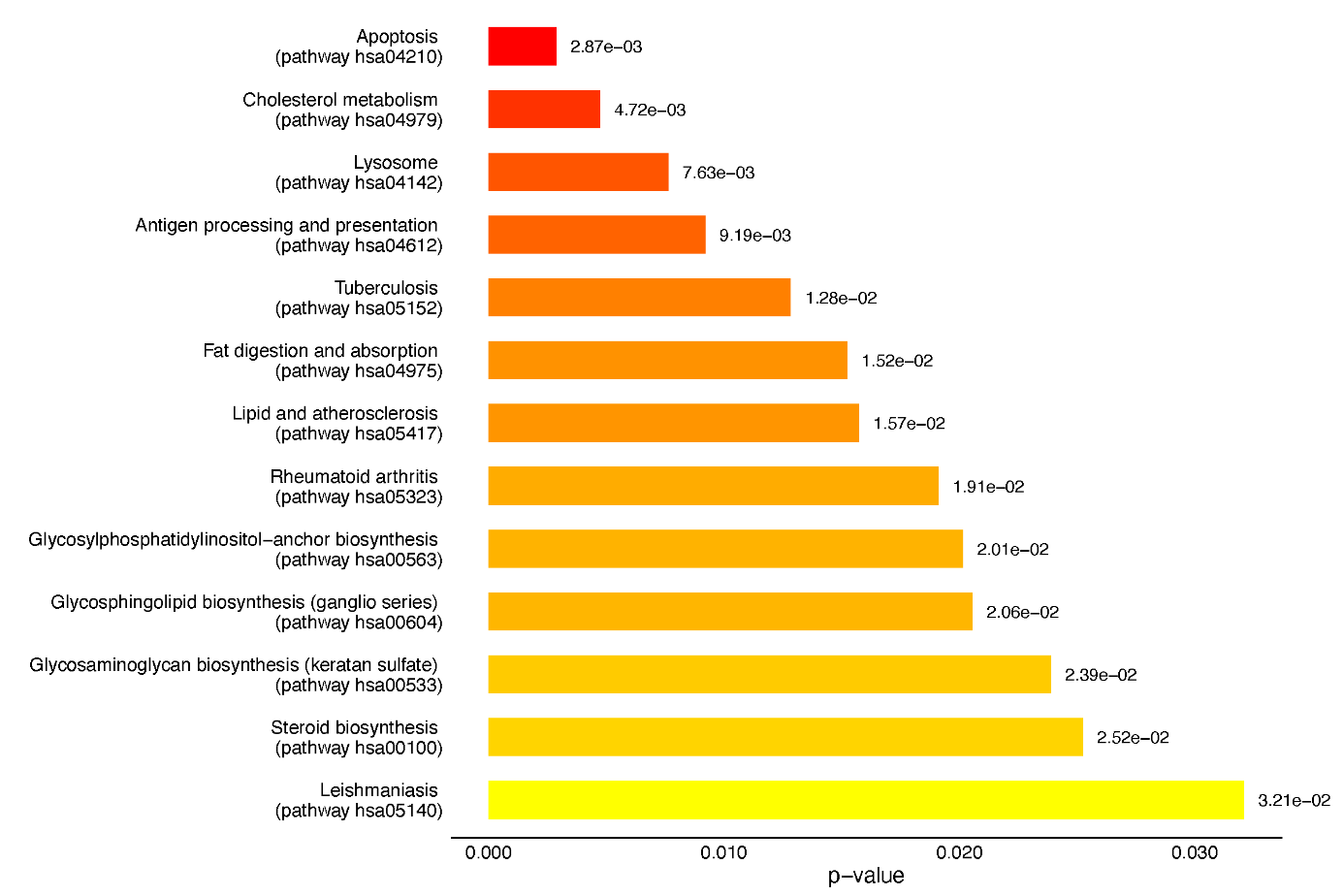
**

**Supplementary Figure S4:** KEGG pathway enrichment analysis of survival-associated serum metabolites. Diffusion-based network analysis illustrating the metabolic pathways significantly impacted by the differentially regulated metabolites between survival groups. The analysis was performed using the FELLA package applied to the KEGG Human database. The resulting sub-networks display interconnected KEGG entities (pathways, modules, enzymes, reactions, and compounds) that are significantly enriched based on the input metabolites (FDR-corrected p-score < 0.10).  For the following parameters, no KEGG id was available, and hence these parameters could not be included in the analysis: 2-Methyl-1,3-propanediol, Albumin, Dimethylmalonic acid, Total protein and the lipoprotein parameters. FDR=false discovery rate; id=identifier; KEGG=Kyoto Encyclopedia of Genes and Genomes.

**
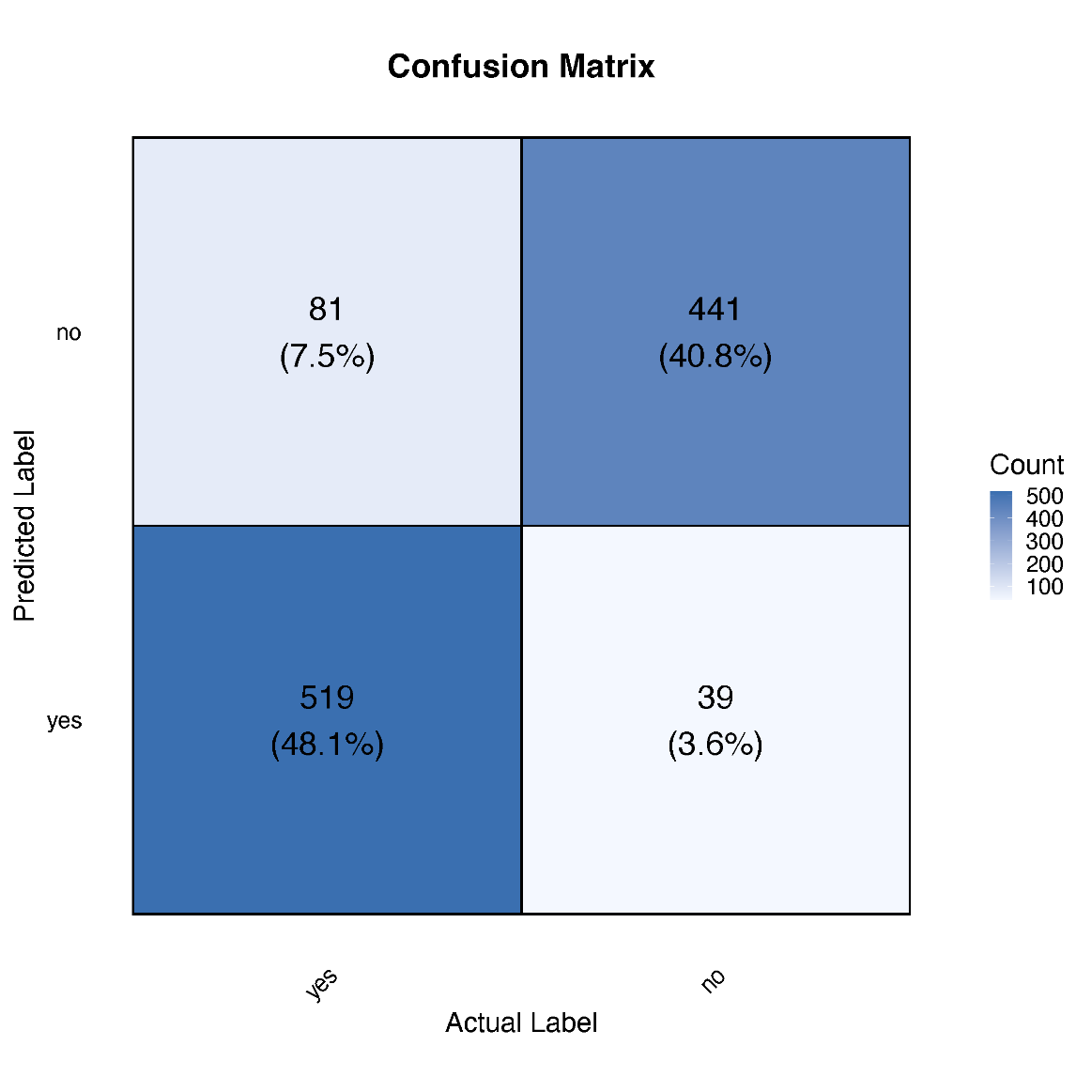
**

**Supplementary Figure S5**: **Confusion matrix of Random Forest model for survival**. Predictions from all cross-validation folds (5 times repeated 10-fold cross-validation) were aggregated, and the optimal classification threshold was derived using Youden's J statistic. The matrix depicts the total number and percentage of correctly and incorrectly classified samples (label 'yes' depicts survival, positive class was 'no').


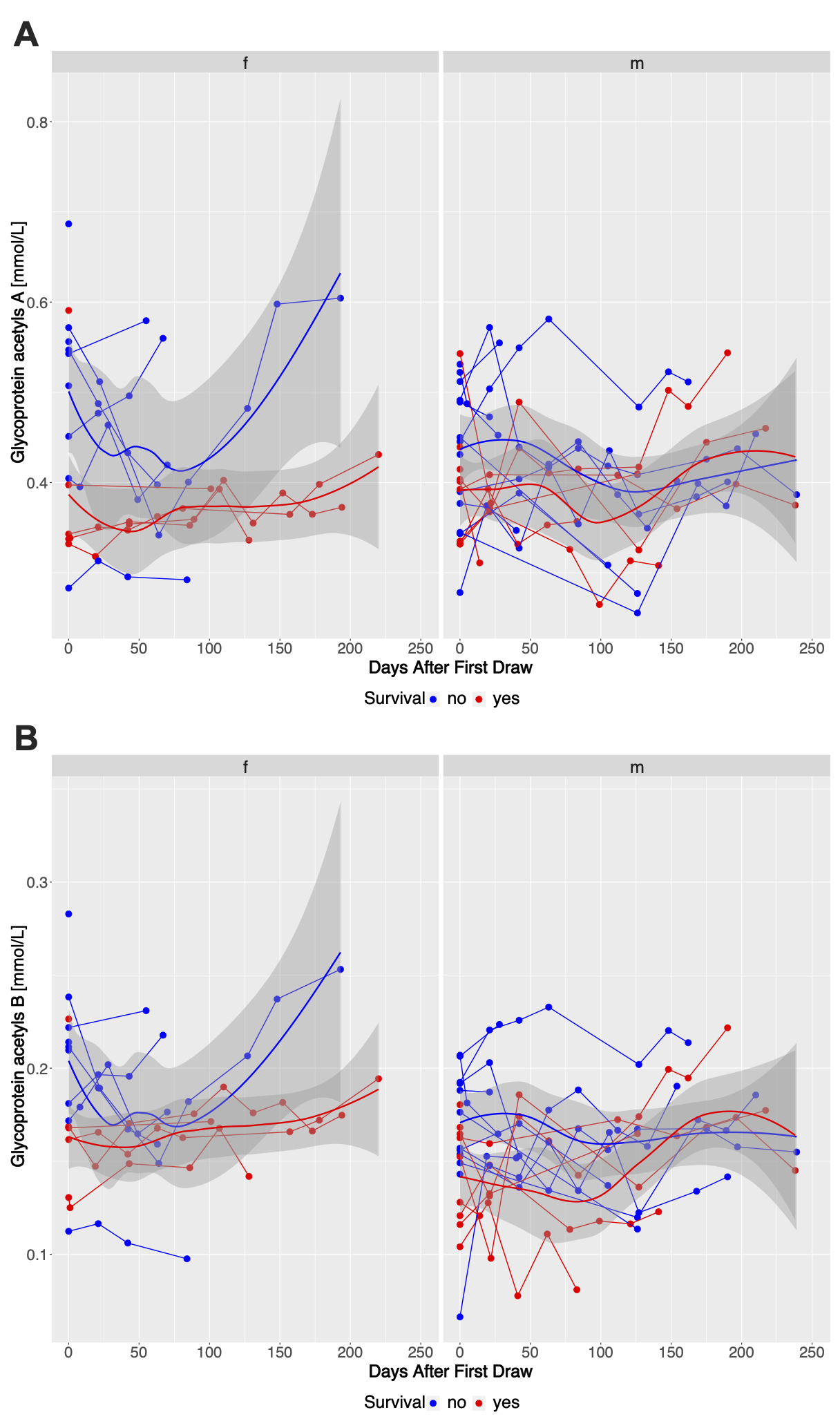


**Supplementary Figure S6: Longitudinal dynamics of selected metabolites over 250 days.** Individual serum values of glycoprotein acetyls A **(A)** and glycoprotein acetyls B **(B)** are plotted, distinguished by sex and survival status. Each panel displays separate plots for females (f, left) and males (m, right). Individual patient data (points and thin lines) and smoothed mean trends (thicker lines with 95% confidence interval shading) are shown for patients who did not survive ('no', blue) and those who survived ('yes', red). f=females; m=males.
